# Template-based C8-SCORPION: a protein 8-state secondary structure prediction method using structural information and context-based features

**DOI:** 10.1186/1471-2105-15-S8-S3

**Published:** 2014-07-14

**Authors:** Ashraf Yaseen, Yaohang Li

**Affiliations:** 1 Department of Computer Science, Old Dominion University, Norfolk, VA 23529, USA

## Abstract

**Background:**

Secondary structures prediction of proteins is important to many protein structure modeling applications. Correct prediction of secondary structures can significantly reduce the degrees of freedom in protein tertiary structure modeling and therefore reduces the difficulty of obtaining high resolution 3D models.

**Methods:**

In this work, we investigate a template-based approach to enhance 8-state secondary structure prediction accuracy. We construct structural templates from known protein structures with certain sequence similarity. The structural templates are then incorporated as features with sequence and evolutionary information to train two-stage neural networks. In case of structural templates absence, heuristic structural information is incorporated instead.

**Results:**

After applying the template-based 8-state secondary structure prediction method, the 7-fold cross-validated Q8 accuracy is 78.85%. Even templates from structures with only 20%~30% sequence similarity can help improve the 8-state prediction accuracy. More importantly, when good templates are available, the prediction accuracy of less frequent secondary structures, such as 3-10 helices, turns, and bends, are highly improved, which are useful for practical applications.

**Conclusions:**

Our computational results show that the templates containing structural information are effective features to enhance 8-state secondary structure predictions. Our prediction algorithm is implemented on a web server named "C8-SCORPION" available at: http://hpcr.cs.odu.edu/c8scorpion.

## Background

An important intermediate step in modeling the three-dimensional structure of a protein is to accurately predict its secondary structures [1]. Most often, the secondary structures are classified into three general states, i.e., helices (H), strands (E), and coils (C). Correspondingly, success of secondary structure prediction is typically measured by the Q3 (3-state) accuracy. Many machine learning methods, including statistics analysis, neural networks, hidden Markov chain, support vector machines, have been developed to predict secondary structures. Correspondingly, there are many secondary structure prediction servers available, including GOR4 [2], PSI-Pred [3], PHD [4], SAM [5], Porter [6], JPred [7], SPINE [8], SSPRO [9], NETSURF [10], and many others. The modern secondary structure prediction servers can generate prediction results with close to 80% Q3 accuracy.

Compared to the general three secondary structure states, the DSSP program [11] has more detailed classifications by assigning secondary structures to eight states, including 3-10 helix (G), α-helix (H), π-helix (I), β-stand (E), bridge (B), turn (T), bend (S), and others (C). The 8-state secondary structures convey more precise structural information than 3-state, which is particularly important for a variety of applications. For example, accurate 8-state secondary structures predictions can restrict the variations of backbone dihedral angles within a small range according to the Ramachandran plots [12] and thus reduce the search space in template-free protein tertiary structure modeling. Moreover, differentiations among 3-10 helix, α-helix, and π-helix in secondary structure prediction aid to assign residues and fit protein structure models in cryo-electron microscopy density maps [13]. Unfortunately, most of the secondary structure prediction software packages or servers only provide 3-state predictions.

**Figure 1:**
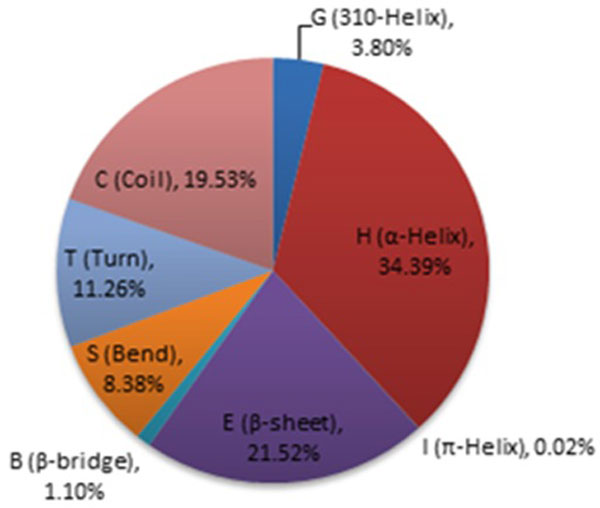
**Distribution of 3-10 helices (G), α-helices (H), π-helices (I), β-sheets (E), β-bridges (B), turns (T), bends (S), and coils (C) in Cull5547**.

To the best of our knowledge, very few methods have been developed for the 8-state secondary structure prediction. Pollastri et al. [9] extended their 3-state prediction method to SSpro8 for 8-state secondary structure prediction. The reported Q8 accuracy of SSpro8 is 62-63% [9]. A more recent prediction method of the 8-state, RaptorXss8, developed by Wang et al [14], has reported 67.9% Q8 accuracy through the use of conditional neural field (CNF) models. Table 1 shows the prediction accuracy of RaptorXss8 on several popularly used secondary structure prediction benchmarks, including CB513, CASP9, Manesh215, and Carugo338. Although nearly 70% Q8 accuracy is achieved, the prediction accuracies of different states vary significantly. In particular, the prediction accuracy of G, I, B, and S are very low, mainly due to the fact of their relatively infrequent appearance in protein data banks (PDB), whose distribution is shown in Figure 1. The low prediction accuracies in these states limit the application of 8-state secondary structure prediction in practice.

**Table 1:** Prediction Accuracy of RaptorXss8 on Benchmarks of CB513, CASP9, Manesh215, and Carugo338.

	Q_G_	Q_H_	Q_I_	Q_E_	Q_B_	Q_S_	Q_T_	Q_C_	Q_8_
CB513	17.54	89.96	0.00	77.68	0.09	15.87	48.02	63.29	65.59

CASP9	20.58	92.90	0.00	81.64	0.00	18.11	51.45	59.37	69.31

Manesh215	18.43	90.22	0.00	79.60	0.32	17.80	51.28	63.73	67.69

Carugo338	19.20	89.91	0.00	79.45	0.44	17.14	50.11	63.36	66.64

Prediction accuracies for 3-10 helices (G), π-helices (I), β-bridges (B), and bends (T) are particularly low due to their low appearance frequencies.

Most current secondary structure perdition methods do not rely on similarity to known protein structures; in other words, these methods are *de novo*, where the secondary structure prediction is based on sequence information only. However, we cannot neglect the fact that many protein sequences have some degree of similarity among themselves. Actually, over half of all known protein sequences have some detectable similarity (higher than 25%) to one or more sequences of known structures [15,16]. Around 75% was reported as the percentage of those newly deposited protein structures in the PDB database showing significant similarity to previous deposited structures. Consequently, taking advantage of structural similarity of proteins with sequence similarity may lead to significant improvement of protein structure prediction. In fact, the latest version of porter [6] has used homology-based templates for 3-state secondary structure prediction [16]. Porter has been reported to achieve prediction accuracy improvement when known structures with >30% sequence similarity are available and even reach theoretical upper bound of secondary structure prediction when such sequence similarity is higher than 50%.

In this paper, we investigate the template-based method for 8-state secondary structure prediction. We extract structural information from known structures of chains with certain sequence similarity to build structural templates. Then, the structural information contained in the templates is incorporated (as features) together with sequence and evolutionary information for neural network training and validation.

In the case where structural information from the structural template is not available for a residue, context-based scores estimating the favorability of that residue adopting a secondary structure conformation in the presence of its neighbors in sequence are used instead. The fundamental idea of the context-based scores is based on the fact that the formation of secondary structure exhibit strong local dependency, particularly, residues in a protein sequence are strongly correlated in different sequence positions in coils, β-sheets, 310 helices, α-helices, and π-helices. We extract statistics to derive context-based scores from a large training data set. These context-based scores are then incorporated as sequence-structure features together with sequence, template, and evolutionary information in neural network training process for 8-state secondary structure prediction.

We test our template-based 8-state prediction method on several popularly used benchmarks including CB513, Manesh215, and Carugo338 as well as the CASP9 targets. The prediction accuracies for the eight states are analyzed.

## Methods

### The protein data sets

We use the protein chain dataset Cull5547 generated by the PISCES server [17] on 10/21/2011 for neural network training and Cull16633 for context-based scores generation. Cull5547 contains 5,547 protein chains with at most 25% sequence identity and 2.0A resolution cutoff, and Cull16633 contains 16,633 protein chains with at most 50% sequence identity and 3.0A resolution cutoff. We eliminate very short chains, whose lengths are less than 40 residues, since the PSI-BLAST program [18] is usually unable to generate profiles for very short sequences, and very large chains whose lengths are greater than 1,000 residues. We also eliminate residue samples with undetermined secondary structures.

Public benchmarks, including CB513 [19], Manesh215 [20], Carugo338 [21], and the recent CASP9 targets [22], that are popularly employed as benchmarks for 3-state secondary structure predictions, are used to benchmark our method in 8-state predictions.

### Template construction

**Figure 2:**
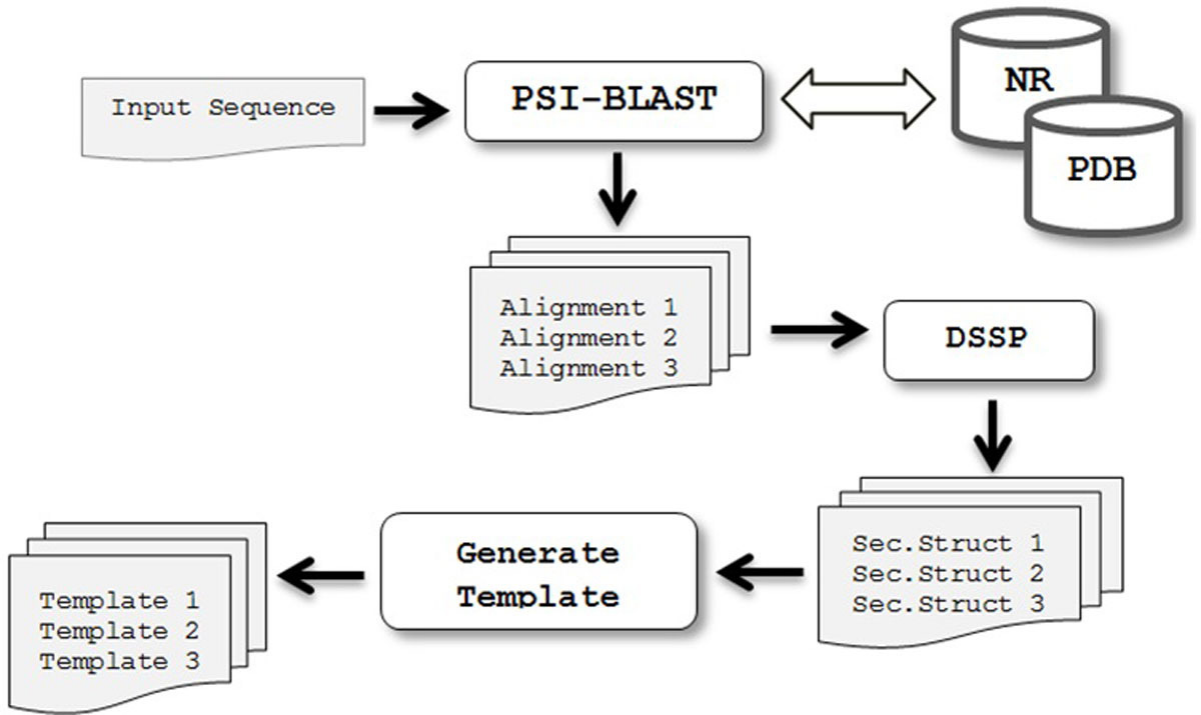
**Construction of Templates**.

Figure 2 illustrates the procedure of constructing structural templates. First of all, for a given protein sequence target, PSI-BLAST is used to search against the NR (Non-Redundant) database with E-value = 0.001 and at most 3 iterations to generate the PSSM (Position Specific Scoring Matrix) data. Then, the PSSM is used to search against the Protein Data Bank (PDB [23]) for alignments with E-value = 10.0. If known structures are available in PDB, their 8-state assignments are determined by the DSSP program and then a structural template is built for the correspondent residue positions. Among the list of templates constructed, we select the top one that is less than 95% sequence similarity, according to PSI-BLAST ranking.

### Encoding

We use a window size of 15 residues for input encodings. Each residue is represented with 20 values from the PSSM (Position-Specific Scoring Matrix) data, 1 extra input to indicate if the residue window overlaps C- or N-terminal, 1 value for degree of similarity, and 8 values for structural information from template or context-based secondary structure scores [24]. Hence, a total number of 450 values are used to describe each residue

**Figure 3:**
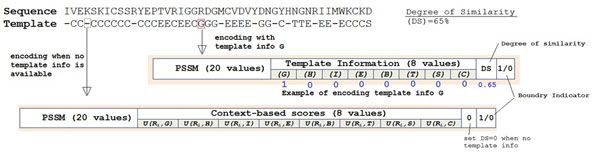
**Encoding for template-based 8-state secondary structure prediction**.

Figure 3 shows an example of encoding residues in a protein sequence. For a residue with available structural information in the template, the corresponding secondary structure state is set to 1 while the other states are set to 0. At the same time, the degree of similarity is set for the sequence similarity. On the other hand, if the structural information for a residue is not available in the template, the degree of similarity is set to zero and the context-based scores are incorporated instead. The context-based scores are statistics-based pseudo-potentials to specify the favorability of a residue adopting a certain secondary structure in its amino acid context [24].

### Context-based scores

The types and conformations of nearby residues play a critical role in secondary structure conformation that a residue may adopt [24]. In particular, the hydrogen bonds between residues at positions *i *and *i *+ 3, *i *and *i *+ 4, and *i *and *i *+ 5 lead to the formation of 3-10-helices, α-helices, and π-helices, respectively. Residues in contacting parallel or anti-parallel β-sheets are connected by hydrogen bonds in alternative positions. Moreover, the formation of interactions within coils beyond nearest neighbors appears not to contribute with statistical significance in determining coil structure [27]. Hence, correlations among residues provide significant information in predicting secondary structure.

In this method, we will extract statistics of singlets $\left(R_{i}\right)$, doublets $\left(R_{i}R_{i+k}\right)$, and triplets $\left(R_{i}R_{i+k_{1}}R_{i+k_{2}}\right)$ residues at different relative positions from protein sequences in Cull16633 dataset. These statistics represent estimations of the probabilities of residues adopting a specific structural state when none, one, or two of their neighbors in context are taken into consideration, respectively.

The observed probabilities of the *i*^th ^residue $R_{i}$ in a singlet $\left(R_{i}\right)$, doublet $\left(R_{i}R_{i+k}\right)$, and triplet $\left(R_{i}R_{i+k_{1}}R_{i+k_{2}}\right)$ adopting a specific structural state ${\text{C}}_{\text{i}}$ are respectively estimated by

$$P_{obs}\left(C_{i}\text{|}R_{i}\right)=\frac{N_{obs}\left(C_{i},R_{i}\right)}{N_{obs}\left(R_{i}\right)},$$

$$P_{obs}\left(C_{i}\text{|}R_{i}R_{i+k}\right)=\frac{N_{obs}\left(C_{i},R_{i}R_{i+k}\right)}{N_{obs}\left(R_{i}R_{i+k}\right)},\text{and}$$

$$P_{obs}\left(C_{i}\text{|}R_{i}R_{i+k_{1}}R_{i+k_{2}}\right)=\frac{N_{obs}\left(C_{i},R_{i}R_{i+k_{1}}R_{i+k_{2}}\right)}{N_{obs}\left(R_{i}R_{i+k_{1}}R_{i+k_{2}}\right)}.$$

Here $N_{obs}\left(C_{i},R_{i}\right)$, $N_{obs}\left(C_{i},R_{i}R_{i+k}\right)$, and $N_{obs}\left(C_{i},R_{i}R_{i+k_{1}}R_{i+k_{2}}\right)$ are the weighted observed number of singlet $\left(R_{i}\right)$, doublet $\left(R_{i}R_{i+k}\right)$, and triplet $\left(R_{i}R_{i+k_{1}}R_{i+k_{2}}\right)$ with $R_{i}$ adopting conformation $C_{i}$ in the protein structure database. $N_{obs}\left(R_{i}\right)$, $N_{obs}\left(R_{i}R_{i+k}\right)$, and $N_{obs}\left(R_{i}R_{i+k_{1}}R_{i+k_{2}}\right)$ are the weighted observed number of singlets, doublets, and triplets. The observed numbers will be calculated as

$$N_{obs}\left(R_{i}\right)=\underset{Protein}{\sum }\underset{j}{\sum }PSSM_{j}\left(R_{i}\right),$$

$$N_{obs}\left(R_{i}R_{i+k}\right)=\underset{Protein}{\sum }\underset{j}{\sum }PSSM_{j}\left(R_{i}\right)*PSSM_{j}\left(R_{i+k}\right),$$

$$N_{obs}\left(R_{i}R_{i+k_{1}}R_{i+k_{2}}\right)=\underset{Protein}{\sum }\underset{j}{\sum }PSSM_{j}\left(R_{i}\right)*PSSM_{j}\left(R_{i+k_{1}}\right)*PSSM_{j}\left(R_{i+k_{2}}\right),$$

$$N_{obs}\left(C_{i},R_{i}\right)=\underset{Protein}{\sum }\overset{C_{j}=C_{i}}{\underset{j}{\sum }}PSSM_{j}\left(R_{i}\right),$$

$$N_{obs}\left(C_{i},R_{i}R_{i+k}\right)=\underset{Protein}{\sum }\overset{C_{j}=C_{i}}{\underset{j}{\sum }}PSSM_{j}\left(R_{i}\right)*PSSM_{j}\left(R_{i+k}\right),\text{and}$$

$$N_{obs}\left(C_{i},R_{i}R_{i+k_{1}}R_{i+k_{2}}\right)=\underset{Protein}{\sum }\overset{C_{j}=C_{i}}{\underset{j}{\sum }}PSSM_{j}\left(R_{i}\right)*PSSM_{j}\left(R_{i+k_{1}}\right)*PSSM_{j}\left(R_{i+k_{2}}\right),$$

where $PSSM_{j}\left(R_{i}\right)$ is the PSSM frequency for residue type $R_{i}$ at the *j*^th ^position of a protein sequence.

Correspondently, the context-dependent pseudo-potentials are generated using the derived statistics of correlations between each residue and its nearby neighbors based on Sippl's potentials of mean force method [25]. According to the inverse-Boltzmann theorem, we calculate the mean-force potential $U_{singlet}\left(R_{i},C_{i}\right)$ for a singlet residue $R_{i}$ adopting structural state ${\text{C}}_{\text{i}}$,

$$U_{singlet}\left(C_{i},R_{i}\right)=-RTln\frac{P_{obs}\left(C_{i}\text{|}R_{i}\right)}{P_{ref}\left(C_{i}\text{|}R_{i}\right)}.$$

Here *R *is gas constant, *T *is temperature, and $P_{ref}\left(C_{i}\text{|}R_{i}\right)$ is the referenced probability. In our method, we will employ the conditional probability approach described in [28] to estimate the referenced probability by

$$P_{ref}\left(C_{i}\text{|}R_{i}\right)=\overset{C_{j}=C_{i}}{\underset{j}{\sum }}N_{obs}\left(C_{j},R_{j}\right)/\underset{j}{\sum }N_{obs}\left(R_{j}\right).$$

Similarly, the mean-force potentials $U_{doublet}\left(C_{i},R_{i}R_{i+k}\right)$ and $U_{triplet}\left(C_{i},R_{i}R_{i+k_{1}}R_{i+k_{2}}\right)$ for residue adopting structural state are

$$U_{doublet}\left(C_{i},R_{i}R_{i+k}\right)=-RTln\frac{P_{obs}\left(C_{i}\text{|}R_{i}R_{i+k}\right)P_{ref}\left(C_{i}\text{|}R_{i}\right)}{P_{ref}\left(C_{i}\text{|}R_{i}R_{i+k}\right)P_{obs}\left(C_{i}\text{|}R_{i}\right)}$$

and

$$U_{triplet}\left(C_{i},R_{i}R_{i+k_{1}}R_{i+k_{2}}\right)=-RTln\frac{P_{obs}\left(C_{i}\text{|}R_{i}R_{i+k_{1}}R_{i+k_{2}}\right)P_{ref}\left(C_{i}\text{|}R_{i}R_{i+k_{2}}\right)P_{ref}\left(C_{i}\text{|}R_{i}R_{i+k_{1}}\right)P_{obs}\left(C_{i}\text{|}R_{i}\right)}{P_{ref}\left(C_{i}\text{|}R_{i}R_{i+k_{1}}R_{i+k_{2}}\right)P_{obs}\left(C_{i}\text{|}R_{i}R_{i+k_{2}}\right)P_{obs}\left(C_{i}\text{|}R_{i}R_{i+k_{1}}\right)P_{ref}\left(C_{i}\text{|}R_{i}\right)},$$

with the corresponding referenced probabilities,

$$P_{ref}\left(C_{i}\text{|}R_{i}R_{i+k}\right)=\overset{\begin{array}{c} C_{j}=C_{i}\\ R_{j+k}=R_{i+k} \end{array}}{\underset{j}{\sum }}N_{obs}\left(C_{j},R_{j}R_{j+k}\right)/\underset{j}{\sum }N_{obs}\left(R_{j}R_{j+k}\right),$$

and

$$P_{ref}\left(C_{i}\text{|}R_{i}R_{i+k_{1}}R_{i+k_{2}}\right)=\overset{\begin{array}{c} C_{j}=C_{i}\\ R_{j+k_{1}}=R_{i+k_{1}}\\ R_{j+k_{2}}=R_{i+k_{2}} \end{array}}{\underset{j}{\sum }}N_{obs}\left(C_{j},R_{j}R_{j+k_{1}}R_{j+k_{2}}\right)/\underset{j}{\sum }N_{obs}\left(R_{j}R_{j+k_{1}}R_{j+k_{2}}\right),$$

respectively.

Then, the context-dependent pseudo-potential for $R_{i}$ will be

$$U\left(C_{i},R_{i}\right)=U_{singlet}\left(C_{i},R_{i}\right)+\underset{k}{\sum }U_{doublet}\left(C_{i},R_{i}R_{i+k}\right)+\underset{k_{1},k_{2}}{\sum }U_{triplet}\left(C_{i},R_{i}R_{i+k_{1}}R_{i+k_{2}}\right).$$

These pseudo-potentials are incorporated as context-based scores representing sequence-structure features in neural network training when structural information from templates is not available.

### Neural network model

**Figure 4:**
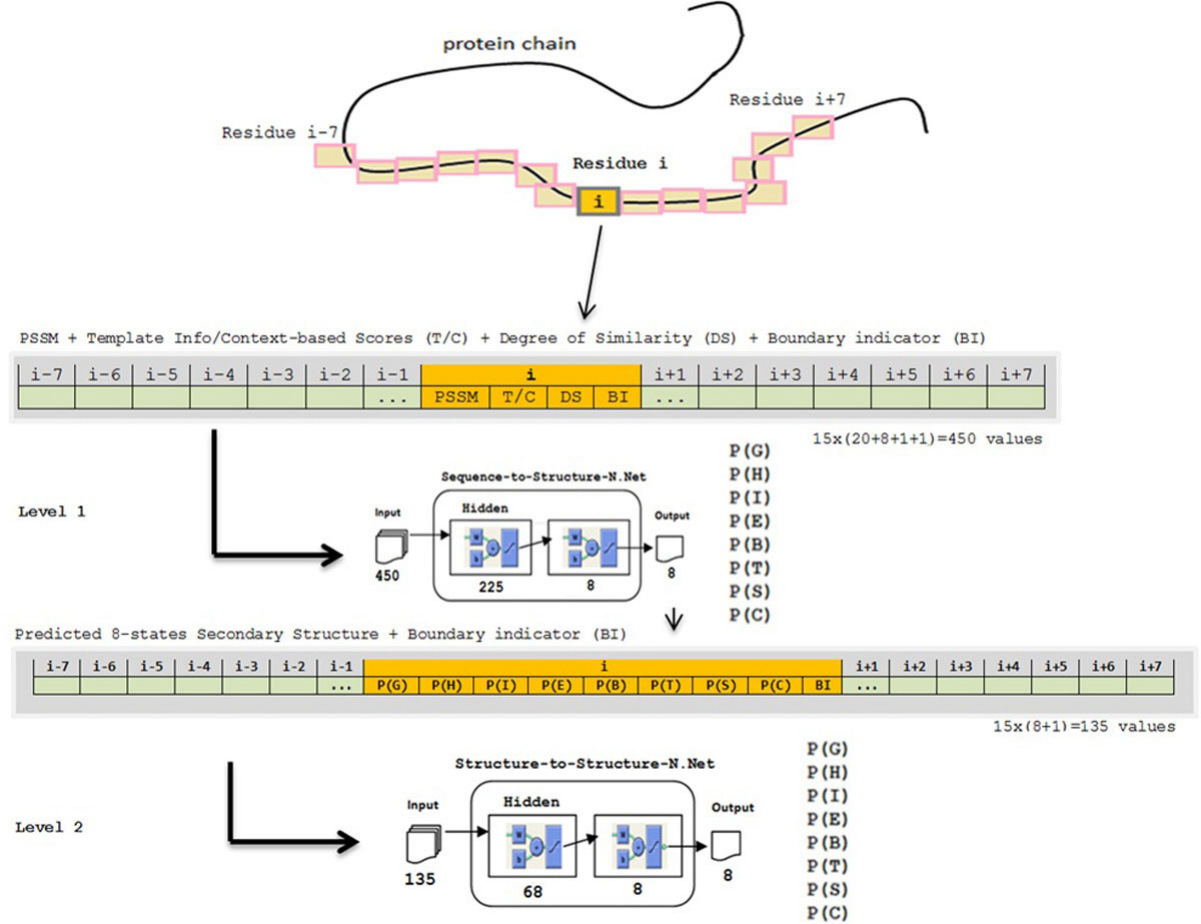
**Two phases of template-based 8-state secondary structure prediction (architecture and encoding)**.

We incorporate two phases of standard feed-forward neural network training for the 8-state secondary structure prediction. The first phase is the primary sequence-structure prediction and the second phase is the structure-structure refinement. The numbers of hidden nodes in the first and second networks are 225 and 68, respectively. Figure 4 shows the encoding diagram and the two-phase neural network architecture. Each neural network is trained to predict the secondary structure state of a residue in the middle of the residue window.

### Performance measures

The prediction accuracy is calculated as the average of the seven prediction scores. We use both Q8 and SOV8 (Segment overlap [26]) scores to measure the qualities of our 8-state secondary structure predictions.

### *N*-fold cross validation

To obtain a reliable estimate of the 8-state secondary structure prediction accuracy, we use 7-fold cross validation on Cull5547. We randomly divide the chains in Cull5547 into 7 subsets with approximately the same size, such that five subsets are used for training, one for testing, and one for validation.

## Results

Upon the selection of the best alignment with similarity less than 95% for all protein chains in the Cull5547 dataset, the final Q8 seven-fold cross validated accuracy after applying the template-based 8-state prediction reaches 78.85%. Table 2 lists the Q8 and SOV8 accuracies of 7-fold cross validation for each state.

**Table 2:** 7-fold cross-validation accuracy in template-based 8-state prediction.

	G	H	I	E	B	S	T	C	Overall
Q_8_	43.99	92.48	0.00	88.30	27.86	43.46	64.18	75.51	78.85

SOV_8_	47.96	95.19	0.00	92.77	27.57	45.32	66.64	71.45	80.10

Table 3 compares the Q8 and SOV8 accuracy of using predictions with and without templates on benchmarks of CB513, CASP9, Manesh215, and Carugo338. Clearly, when homology structural information is available, the 8-state prediction accuracy is significantly improved. It is also interesting to find that when structural templates are used, the 8-state prediction accuracy improvement in CASP9 is much less than the other benchmark sets. This is due to the fact that in the CASP9 experiment, targets are deliberately selected to have relatively low similarity to sequences with existing structures in PDB.

**Table 3:** Comparison between 8-state predictions with and without template on CB513, CASP9, Manesh215, and Carugo338.

	Q_8_		SOV_8_	
	**No-Template**	**With-Template**	**No-Template**	**With-Template**

CB513	67.22	79.39	67.66	80.64
CASP9	71.54	76.36	73.47	78.15
Manesh215	69.71	81.10	70.79	82.99
Carugo338	68.44	80.39	69.50	81.95

**Figure 5:**
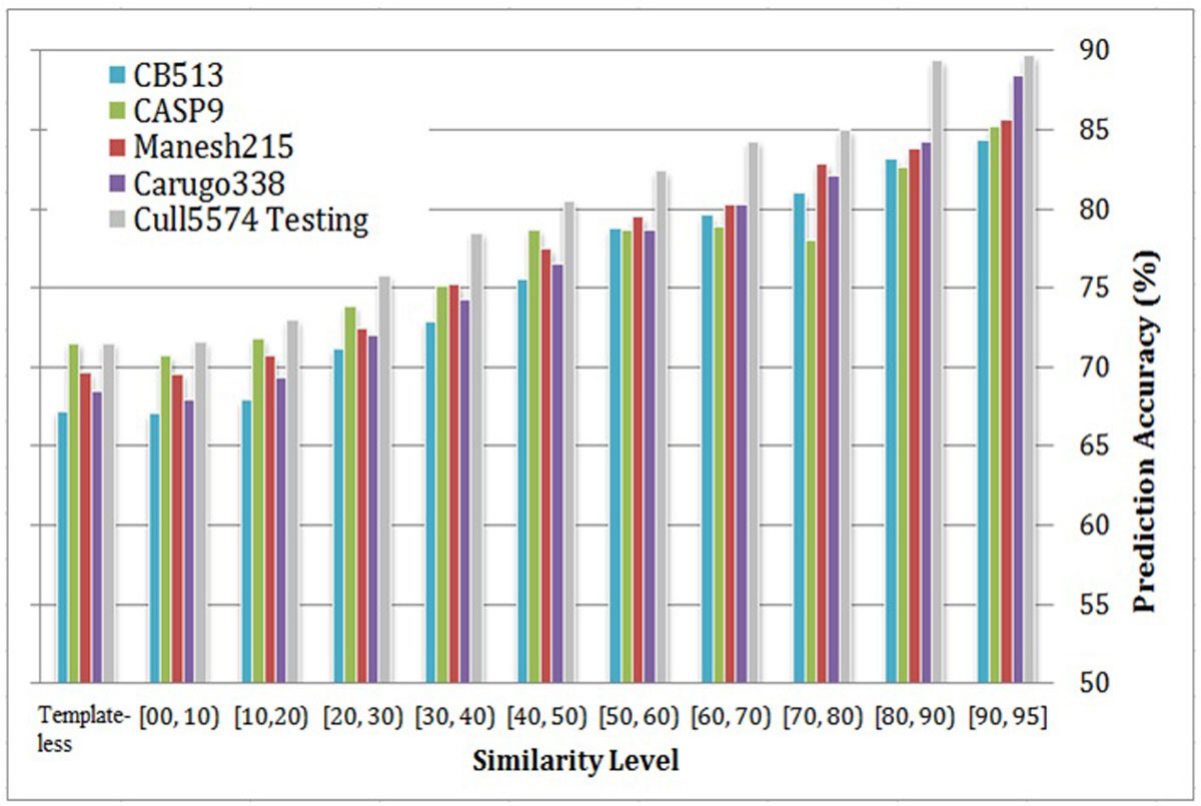
**Distribution of 8-state secondary structure prediction accuracy (Q8) as a function of sequence similarity- the first group of bars corresponds to template-less predictions**.

Figure 5 shows the distribution of the prediction accuracy as a function of sequence similarity in levels in CB513, CASP9, Manesh215, Carugo338 as well as Cull5574 in cross-validation. Without surprise, the better templates with higher sequence similarity level, the more accurate the prediction results are. More importantly, even templates with only 20%~30% sequence similarity can improve the prediction accuracy by near 5% in various benchmark sets compared to predicted results without templates.

**Figure 6:**
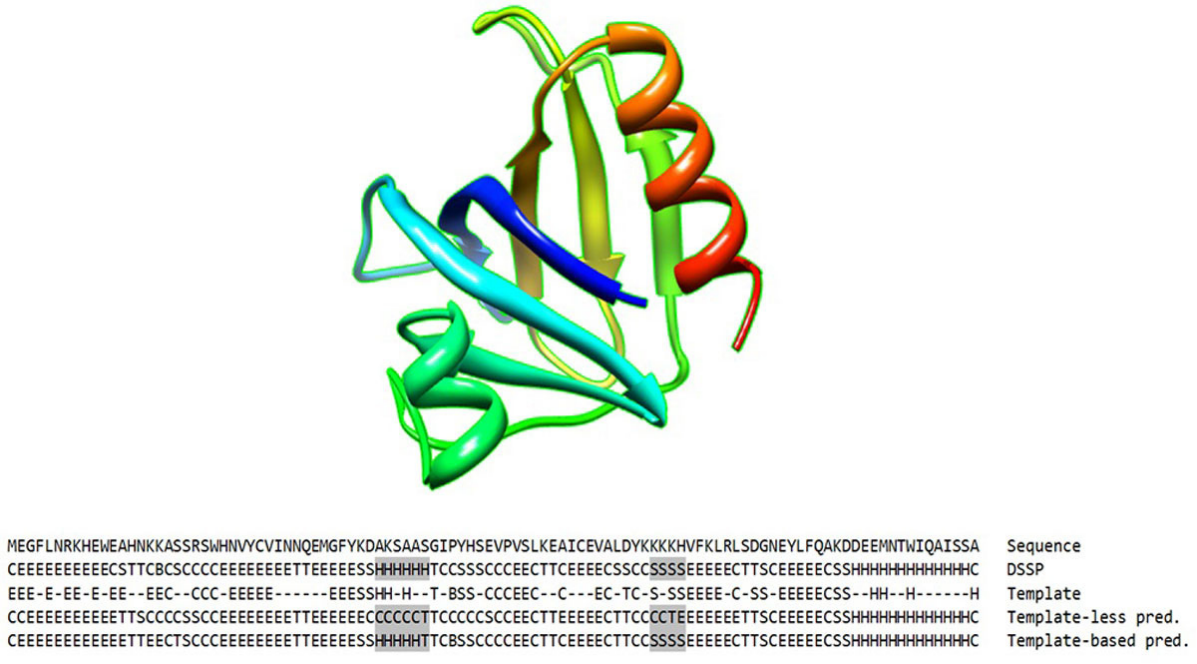
**Comparison between template-less and template-based predictions on 1BTN chain A**.

Figure 6 uses the A chain of protein 1BTN as an example to demonstrate the effectiveness of template-based 8-state secondary structure prediction. Prediction without template has 73.6% Q8 accuracy. The best template found in PDB has 61% sequence similarity. Under the guidance of the structural template, the mispredicted helix segment and bend segment in template-less prediction (highlighted in Figure 6) are corrected, which leads to overall 89.6% Q8 accuracy.

## Discussion

As shown in Table 1, the prediction accuracies for different states vary largely due to the very unbalanced appearing frequencies of the eight states in protein structures. In this paper, we are particularly interested in the effectiveness of structural templates in improving the prediction accuracies of those states with low accuracy in prediction without templates. From Cull5547, we create five subsets of chains that have structural templates with similarity level in intervals of (0%, 10%), (10%, 20%), (20%, 40%), (40%, 70%), and (70%, 95%), respectively. Then, 7-fold neural network trainings are carried out for each subset and the average cross validation prediction accuracy for each state is reported in Table 4.

**Table 4:** Comparison of 7-fold cross validation prediction accuracies in eight states when templates with different sequence similarities are used.

	(0, 10]	(10, 20]	(20, 40]	(40, 70]	(70, 95]
# of chains	4,426	4,215	3,204	1,437	1,133
Q_H_	92.05	92.70	93.60	94.97	95.94
Q_G_	22.07	23.93	35.09	55.03	69.44
Q_I_	0.00	0.00	0.00	0.00	0.00
Q_E_	83.37	84.53	86.59	90.16	93.61
Q_B_	1.53	3.59	7.24	22.30	44.26
Q_T_	53.35	55.34	60.89	69.66	77.06
Q_S_	22.83	26.41	35.19	54.09	73.40
Q_C_	66.55	67.84	71.81	79.56	86.80
Q_8_	71.33	73.01	76.29	82.11	88.01

For α-helices (H), the prediction accuracy using templates with very low sequence similarity (0%, 10%] is already rather high (92.05%), mainly because there are sufficient number of α-helix samples available and the formation of α-helix is mainly result from local interactions. Anyway, the structural templates help refine the α-helix predictions with slight accuracy improvements. When structural templates with 40% or better similarity are available, the prediction accuracy of β-sheets (E) is also improved to above 90%, reaching the theoretical upper bound in secondary structure prediction. 40%+ similarity templates also significantly improve the accuracies of 3-10 helices (G) and bends (S) from 20%+ to 50%+. Similar but not as significant improvements are found in turns (T) and coils (C). However, the prediction results for bridges (B) and π-helices (I) are disappointing. Only when templates with very high similarity (>70%) are available, we can obtain 44% prediction accuracy in bridges (B). The prediction accuracy for π-helices (I) is still 0%. This is mainly due to the facts that π-helices are extremely rare (0.02%) and π-helices (I) are often misclassified into α-helices (H).

## Conclusions

We describe a template-based approach to enhance 8-state secondary structure prediction accuracy in this paper. Our computational results show that the secondary structure templates, even obtained from sequence with only 20%~30% sequence similarity, can help improve the 8-state prediction accuracy. Overall, 78.85% Q8 accuracy and 80.10% SOV8 accuracy are achieved in 7-fold cross validation. The effectiveness of using structural information in templates has been demonstrated on popular benchmarks including CB513, CASP9, Manesh215, and Carugo338. More importantly, when good templates are available, the prediction accuracy of less frequent secondary structure states, such as 3-10 helices, turns, and bends, are highly improved, which are suitable for practical use in applications.

A webserver (C8-Scorpion) implementing 8-state secondary structure prediction is currently available at http://hpcr.cs.odu.edu/c8scorpion.

## Competing interests

The authors declare that they have no competing interests.

## Authors' contributions

YL conceived the context-based scoring method. AY implemented the method and carried out the computation. AY and YL performed the result analysis. Both authors read and approved the final manuscript.

## Declarations

Publication charges for this work were funded by NSF grant 1066471 to YL.

This article has been published as part of *BMC Bioinformatics *Volume 15 Supplement 8, 2014: Selected articles from the Third IEEE International Conference on Computational Advances in Bio and Medical Sciences (ICCABS 2013): Bioinformatics. The full contents of the supplement are available online at http://www.biomedcentral.com/bmcbioinformatics/supplements/15/S8.
